# Towards Efficient Function Optimisation with Quantum–Classical Hybrid Search

**DOI:** 10.3390/e28101060

**Published:** 2026-09-26

**Authors:** Mansur Ziiatdinov, Fedor Levkovich-Maslyuk, Emmanuel M. Pothos

**Affiliations:** 1 Department of Mathematics and Computer Sciences, Physical Sciences and Earth Sciences, University of Messina, Viale Ferdinando Stagno d’Alcontres, 31, 98166 Messina, Italy; 2 Centre for Mathematical Science, City St George’s, University of London, Northampton Square, London EC1V 0HB, UK; fedor.levkovich-maslyuk@citystgeorges.ac.uk; 3 Department of Psychology, City St George’s, University of London, Northampton Square, London EC1V 0HB, UK; emmanuel.pothos.1@citystgeorges.ac.uk

**Keywords:** quantum computing, continuous function optimisation, Grover’s algorithm, Newton–Raphson’s method

## Abstract

Finding the global minimum of a function with many local extrema is well known to be a challenging computational task. We propose a hybrid quantum–classical algorithm for this optimisation problem, based on a combination of Grover quantum search and the classical Newton–Raphson method. The approach is tailored to situations with a large number of false minima. The main idea is to use quantum search to efficiently locate the vicinity of the true optimum, after which a classical algorithm can solve the problem in only a few iterations. We specifically address the nontrivial question of choosing the discretisation of the function domain, which is critical for quantum approaches to optimisation of continuous functions. While we do not expect that this method will always be superior, we identify a paradigmatic case of a function for which we demonstrate that, remarkably, our hybrid method quadratically outperforms a purely classical approach (measuring performance by the number of function/oracle calls). We evaluate key performance metrics of the algorithm in numerical experiments. We furthermore discuss its possible extensions as well as the potential relevance of hybrid approaches in the context of quantum cognition.

## 1. Introduction

One of the most important results in quantum computing is Grover’s algorithm, showing how the identification of a target in unstructured search can be done with $O(\sqrt{N})$ iterations [1], instead of the classical requirement of O(N) iterations. Since its original development, variants of this algorithm have been proposed for a number of tasks, including function optimisation, the subject we focus on in this paper. The importance of this application comes in particular from its central place in modern machine learning approaches.

In general, there has been much excitement regarding the transformative potential of quantum computing (for example, the UK has clearly highlighted quantum technologies as a priority research area, see e.g., [2]), and one tantalising possibility is that quantum computing could facilitate deep neural network training. This is a key technological challenge currently, because of the immense energy needs to train and run such models with classical computing methods [3]. As just one example of how quantum computing could impact neural network training, in [4] a quantum neural network was developed, though this model is currently best suited to problems specific to quantum mechanics.

Optimism about possible quantum computing benefits is suppressed by lack of clarity about how to harness quantum computing power in many practical situations (e.g., see the review of Schuld et al. [5]). For example, one problem in such applications is that to evaluate a candidate solution, one needs to inspect this solution, but doing so typically destroys any quantum resources and breaks the quantum computation. Researchers have been exploring creative and inspired ways to circumvent such limitations. For example, a proposal from [6], concerning learning multivariate joint probability distributions, was based on a ‘copula’, which encapsulates the relationship between two variables. This copula can be mapped onto a quantum entangled state, and it is in this way that quantum computation can be utilised. There are many other problems too, for example, regarding noise resistance. There is an extensive and sophisticated literature, which we do not attempt to consider here. Rather, our goal is to explore a way to mitigate some of the challenges associated with quantum computing, through a particular proposal for combining quantum and classical approaches.

We develop our proposal in the context of function optimisation, as this is a problem of wide applicability and interest (including in relation to neural networks). One natural approach that has been explored recently is to try and extend Grover’s algorithm to this task, with the intuitive adaptation that the search space in Grover’s algorithm corresponds to the values of the function of interest. In Grover’s standard algorithm, each application of the oracle just changes the phase of the target. In Benchasattabuse et al.’s extension [7], the phase changes in a way proportional to the value of some criterion function. Such methods work differently depending on the granularity required for the domain of the function to be optimised. This is because a larger domain entails more Grover iterations before it can be assumed that the algorithm is close enough to the solution. Note, it may appear that discretising continuous problems is a problematic simplification, though there are ways to adapt these ideas to continuous problems (e.g., see [8] for a continuous optimisation algorithm, which can recover Grover when discretised).

Developing applications of Grover’s algorithm to optimisation problems has progressed through several key stages and required a number of nontrivial insights. First, in Grover’s algorithm, it is important to know how many iterations are needed, since deviating from this optimal number will decrease the probability of success. Requiring knowledge of this optimal number can be a tricky limitation. Considering purely quantum approaches first, Boyer et al. [9] derived an estimate for the required number of iterations for the case with an arbitrary number of targets in the search domain. They also introduced a way to make the algorithm work even when the number of targets is unknown: their suggestion was to perform some number of iterations, measure the resulting superposition, check whether the target has been correctly identified and, if not, repeat with a greater number of iterations. Subsequently, Durr and Hoyer [10] applied this idea to the problem of identifying the global minimum of a finite list, with a method where the Boyer et al. [9] algorithm is applied repeatedly to produce increasingly better estimates of the global minimum (each application involves an improved candidate for what the global minimum could be). In [11], Baritompa, Bulger and Wood introduced a unified framework for these ideas, Grover Adaptive Search, and extended some of the relevant proofs. Next, the first hybrid quantum–classical algorithm we could find in this context is due to Lara et al. [12]. They proposed a hybrid search algorithm for global minimisation. It starts with a classical algorithm to find a local minimum, then reverts to the Grover Adaptive Search [11] to identify a possible improvement in order to try to escape from this local extremum. This sequence of classical optimisation followed by a quantum routine is then iterated several times.

Lara et al.’s insight in [12]—that there may be synergy between classical and quantum optimisation and search algorithms—is the main inspiration for the present work. Classical and quantum optimisation have complementary strengths: classical optimisation works very efficiently when we are in the vicinity of the target. Clearly, if we start near the global minimum, then gradient descent or Newton–Raphson methods can converge to the desired solution very quickly. The problem with such classical methods is that they get easily stuck in local minima; a typical approach to avoid this problem is to restart the method several times, with random starting points. Grover search excels for unstructured problems and so offers an alternative, potentially more efficient approach to the local minima problem: instead of using several random starting points as a way to avoid local minima, we propose to employ Grover-based optimisation with the hope of identifying the region where the global minimum is located. Once we are in this region, instead of continuing with Grover (which is blind to the structure of the function and is complicated by the requirement of knowledge of the appropriate number of iterations), we switch to the usual Newton–Raphson method.

More concretely, we extend Lara et al.’s work [12] in two ways: we address the question of how to discretise the domain of the function, and we also compare the resulting hybrid quantum–classical method with a purely classical approach. The choice of discretisation is in fact a crucial step in order to apply Grover-like algorithms–for which the input must be a *discrete* array–to the optimisation of a *continuous* function. While [12] assumed from the start that the domain was discrete, here we start with a usual, purely continuous function and aim to make a fair comparison of our hybrid approach with standard classical optimisation. Recently, Buczkowski et al. [13] also proposed a quantum-accelerated version of a classical algorithm for finding roots of a polynomial, but they did not consider the effects of discretisation on quantum efficiency relative to a matched classical model. As another example, another application of Grover involving polynomials is Sun et al.’s work [14] concerning a cryptographic problem, but the problem is again specified in a discrete domain. Beyond these previous works, of course, Grover search and quantum algorithms based on Grover search have been applied in many areas. However, many of these areas, such as hash collision production, SAT solving, and database search, are discrete problems, so there is no discretisation issue in these applications. It is only with continuous problems that the tension between discretisation and cost (in terms of function queries) arises.

In principle, the quantum algorithm’s running time will increase significantly with many discretisation points. This raises the empirical question of whether the result will be faster, compared to a purely classical approach, which iterates over random starting points but does not require any discretisation of the function. Despite this possible obstruction, we find that, remarkably, there are important regimes in which the quantum–classical hybrid outshines a purely classical method. The goal in this work is to provide an existence demonstration that there are some functions for which the hybrid method excels (acknowledging that there will be many other functions for which this will not be the case). Namely, we focus on functions with many local minima, where classical algorithms frequently get stuck and thus need many runs to find the true minimum. Crucially, we find that in such cases one can choose the discretisation of the domain in a way that it has sufficiently *many* points to distinguish the global from the local minima, while also having sufficiently *few* points for the Grover quadratic speedup to provide an advantage over a purely classical routine. We discuss in detail the nontrivial balance of parameters leading to this outcome in our examples.

Apart from possible optimisation efficiency, a potential advantage of quantum–classical hybrid algorithms is that quantum computation is currently rather costly and, even more importantly, highly prone to errors. Yet on shorter computation timescales, even current or near-future quantum hardware has the potential to work effectively, making it important to explore approaches that use quantum computation to enhance classical algorithms, an example of which we study here.

In summary, we think there are good reasons to explore quantum–classical hybrid algorithms. Foremost, our goal is to examine the possibility that in such algorithms the unique strengths of quantum and classical computation can be brought together, while their specific problems are mitigated, as recognised for optimisation problems already by Lara et al. [12].

**Summary of main results.**

Our main contribution is a hybrid quantum–classical optimisation algorithm that combines a single run of Grover adaptive search with a final Newton–Raphson refinement. The key technical issue we address is the discretisation of a continuous function for a Grover-type procedure: we confirm numerically that, for functions with many local minima, it is sufficient to sample the domain with cM points, where *M* is the number of minima and *c* can be chosen as a constant independent of *M*. For the one-dimensional Rastrigin function, the numerical tests indicate that values around c≈24 already make the search reliable, and we use $c_{\mathrm{opt}}=32$ in the experiments. With this choice, the hybrid method exhibits the expected square-root scaling in the number of oracle calls, whereas a purely classical multi-start Newton method scales linearly with the number of minima. Empirically, the hybrid procedure begins to outperform the classical one at around M≈32, while retaining a high success rate under a realistic stopping rule (performance is quantified in terms of function queries). We also consider in less detail a second example, concerning sums of sine functions. While the relevant parameters were somewhat different, it was possible to identify a reasonable advantage of the hybrid method across the investigated range regarding the number of minima. Finally, we perform a theoretical analysis of the necessary conditions for the hybrid algorithm to succeed.

## 2. Background and Definitions

### 2.1. Notation

Let us denote by [N]≔{0,1,…,N−1} the set of positive integers not exceeding *N*. We also denote $|i\rangle |≔b_{1}\rangle ⊗|b_{2}\rangle ⊗\ldots ⊗|b_{w}\rangle$, where $\bar{b_{1}b_{2}\ldots b_{w}}$ is the binary expansion of the number *i*; the width *w* of the number can usually be inferred from the context.

### 2.2. Quantum Search Algorithms

Let us first briefly review the main features and variations of Grover’s search algorithm, as they will be at the core of the present optimisation approach.

The *unstructured search problem* over a set of *N* elements seeks an element $x^{*}$ that satisfies some conditions. More formally, let there exist a Boolean function f:[N]→{0,1} such that the target states *S* satisfy f(x)=1. This function is encoded as an oracle–a gate $U_{f}$ that flips the phase of all elements that satisfy the condition:

$$U_{f}|x\rangle =\left\{\begin{array}{cc} -|x\rangle , & \;\mathrm{if}\;f(x)=1,\\ |x\rangle , & \;\mathrm{otherwise}. \end{array}\right.$$

The goal is to output an element $x^{*}\in S$ such that $f(x^{*})=1$, using as few quantum queries to $U_{f}$ as possible.

One of the fundamental quantum algorithms–Grover’s algorithm–solves the unstructured search problem with $O(\sqrt{N/M})$ query complexity, providing a quadratic speedup over the classical lower bound of Ω(N/M), where *M* is the number of solutions to f(x)=1 (marked items) [1,9,15]. It starts from the uniform superposition of states $|\psi_{0}\rangle =\frac{1}{\sqrt{N}}\sum_{i=0}^{N-1}|i\rangle$ and performs several iterations of the Grover operator $G≔DO_{f}$ that combines the oracle $O_{f}$ and the diffusion operator $D=|\psi_{0}\rangle \langle \psi_{0}\left|-2|0\rangle \langle 0\right|$. The probability of measuring any of the solutions $x^{*}$ after *r* iterations is then equal to:

(1) $$\mathrm{Prob}\left[MG^{r}|\psi_{0}\rangle =x^{*}\right]=\sin^{2}\left((2r+1)\theta \right),$$

where $\sin\theta =\sqrt{M/N}$.

We will be mainly interested in the application of this algorithm to optimisation tasks. The *optimisation problem* over an array *A* of *N* elements seeks the minimal element. Without loss of generality, we can assume that A⊆[N]. As already briefly mentioned, the quantum Dürr–Høyer algorithm [10] solves this problem by iteratively refining an upper bound of the minimum value using Grover’s search. Baritompa, Bulger, and Wood [11] improved the analysis, suggested a better value for the parameter λ that controls the number of iterations over several Grover runs, and proved that in their approach the expected number of queries to the oracle representing *A* is bounded above by $2.46\sqrt{N}$. Their algorithm variation is described as Algorithm 1.
**Algorithm 1** BBW algorithm.
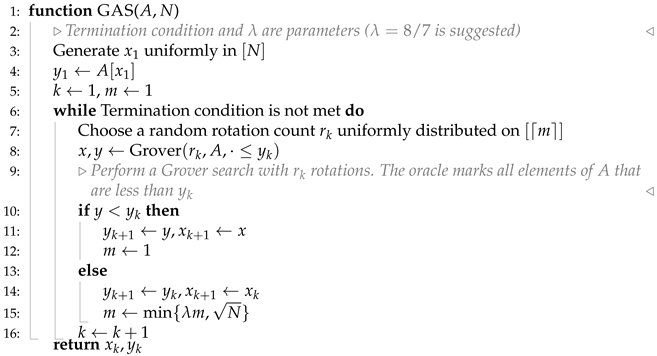


Lara et al. [12] suggested combining Grover adaptive search (Algorithm 1) with a classical optimisation procedure, and refining the minimum value using a classical method after running the quantum procedure, with the quantum and classical regimes following one another multiple times. They also performed numerical experiments comparing the efficiency of different classical procedures inside this hybrid approach. At the same time, they did not address the nontrivial question of discretisation choice or the closely related question of a comparison with a *purely* classical approach. It is these points that will be the main focus of exploration in the present work.

To highlight the key features of different approaches to quantum or hybrid optimisation, we summarise them in Table 1.

### 2.3. Newton–Raphson Method

A standard classical optimisation approach, which will be a part of our hybrid algorithm, is to find zeros of the function’s derivative using the Newton–Raphson method [16]. Newton–Raphson is an iterative procedure to approximate the zeros of a function f(x). Given an initial approximation $x_{0}\in R$, the sequence $\left\{x_{n}\right\}$ is generated by the recursive relation:

(2) $$x_{n+1}=x_{n}-\frac{f(x_{n})}{f^{\prime }\left(x_{n}\right)}$$

where $f^{\prime }\left(x\right)$ denotes the first derivative of *f*. This scheme exploits the first-order Taylor expansion of *f* around $x_{n}$. Under the assumption that $f^{\prime }\left(x_{n}\right)\neq 0$, the method iteratively refines the estimate of the root, converging quadratically if $x_{n}$ is sufficiently close to the true root. In the optimisation context, we apply this method not to the unknown function itself but rather to its derivative, thus finding its zeros, which correspond to locations of the extrema of the original function.

### 2.4. Local Minima and Convergence

In order to formalise theoretical conditions for the algorithm, we need the following definitions.

**Definition** **1.** *Function f:[a,b]→R has a* local minimum *at point $z_{0}\in \left[a,b\right]$ if there exists a neighbourhood $U\left(z_{0}\right)\subset \left[a,b\right]$ of $z_{0}$, such that for all points in the neighbourhood $x\in U\left(x_{0}\right)\setminus \left\{x_{0}\right\}$, $f\left(x_{0}\right)<f\left(x\right)$.*

**Definition** **2.** *A local minimum z∈[a,b] has a* region of convergence of radius *rif for any point x∈(z−r,z+r), Newton’s method starting at the point x converges to the minimum z.*

### 2.5. Rastrigin Function

As the main example to explore our proposal, we will use the Rastrigin function [17], a standard test function frequently employed for the evaluation of optimisation algorithms. It is defined over *n* variables as:

(3) $$f_{\mathrm{Rastrigin}}\left(x\right)=An+\sum_{i=1}^{n}\left[x_{i}^{2}-A\cos\left(2\pi x_{i}\right)\right],$$

where *A* is a positive constant. Here we will typically take A∼100, which ensures the function has sufficiently many local minima. The numerous minima arise due to the periodic cos terms, creating a complex search landscape for optimisation algorithms. We will focus here on the 1-dimensional case. We will denote the Rastrigin function as $f_{R1}:R\to R$, which explicitly reads:

(4) $$f_{R1}\left(x\right)=A+x^{2}-A\cos\left(2\pi x\right).$$

This one-dimensional variant retains the periodic oscillations and the global minimum at the origin, making it a useful and nontrivial test case for search strategies.

### 2.6. Random Sine Sum Functions

Consider a random function f(x) that has the following form:

(5) $$f\left(x\right)=\sum_{i=1}^{K}a_{i}\cos\left(\omega_{i}x+b_{i}\right),$$

where $a_{i}$ are chosen from a uniform distribution on [−1;1], $b_{i}$ are chosen from [0;2π] and $\omega_{i}$ are chosen from $\left\{0,1,\ldots ,\omega_{\max}\right\}$. Note that the frequencies are integers, so the function has a period of 2π. Therefore, we can have an upper bound on the number of minima by choosing the appropriate domain.

## 3. Algorithm and Results

### 3.1. Hybrid Algorithm

Let us now describe in detail our proposed algorithm. We give its explicit form below as Algorithm 2.

As noted, since a key part of the algorithm is the Grover-like search, we will need to discretise the function’s domain. Therefore, the discretisation grid size will be one of the key parameters. If the approximate count *M* of minima is known, the discretisation step will be chosen such that we sample ∼*cM* points for some *c*. Note that—even though for a candidate unknown function this information would not be available—*M* could be estimated from a relevant class of functions. In any case, the main idea is that Grover’s algorithm can find a point that is close to the global minimum; subsequently, the Newton–Raphson method can be employed to descend from there to the actual true minimum. We will shortly see that indeed choosing ∼*cM* points is enough to ensure that the Grover algorithm typically does get us close enough to the true minimum. As a result, the hybrid algorithm performs $O(\sqrt{cM})$ function evaluations/oracle calls (neglecting the small number of further evaluations needed for the final classical run). This should be compared with the pure classical method, which would require ∼*M* function evaluations on average, as starting from a random point will typically result in getting stuck in local minima. As a result, we should get a quadratic advantage over the purely classical method, for large enough *M*.
**Algorithm 2** The proposed hybrid quantum–classical optimisation algorithm.
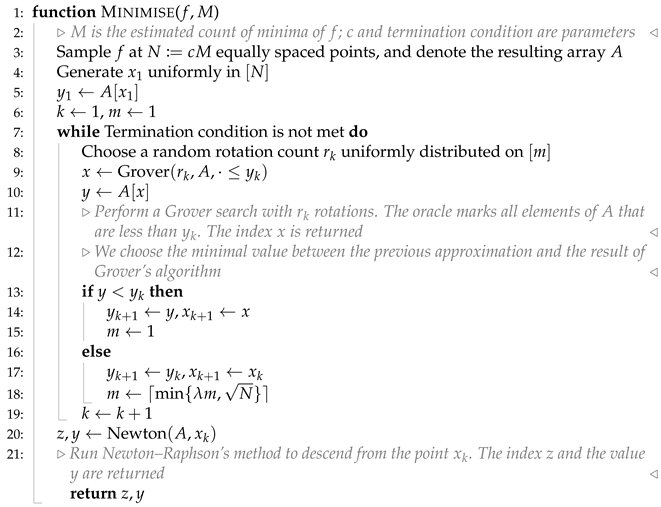


Let us now discuss some analytic estimates for the discretisation parameters. We expect the Newton–Raphson method to converge to the true minimum, as long as the starting point is close enough to it, compared to the width of the minimum. We can get a rough parametric estimate for the latter, which we denote by Δx, by assuming the difference of values of the function at neighbouring minima is ∼1, i.e., $O(M^{0})$ (as is the case for our function). Then, Taylor-expanding around the minimum $x_{0}$ gives $f^{\prime \prime }\left(x_{0}\right){\left(\Delta x\right)}^{2}∼1$, so $\Delta x∼\sqrt{1/f^{\prime \prime }\left(x_{0}\right)}$. If the discretisation step is *h*, roughly speaking we should require h≲Δx. In our case, for a function with *M* minima, the range of *x* is of order ∼*M*, so with cM grid points we will have h∼1/c. Having fixed the original function and taking *M* large, the value of $f^{\prime \prime }\left(x_{0}\right)$ is fixed. Subsequently, the condition Δx≲h translates to (again as a rough parametric estimate) $\sqrt{f^{\prime \prime }\left(x_{0}\right)}≲c$, which will be guaranteed for large enough *c*. Let us emphasise that this argument implies that after we find a good enough value of *c*, we can use it for any number of minima *M*; in other words, we have $c∼O(M^{0})$. As a result, we see that indeed it should be possible to keep only cM discretisation points, with some constant *c*, which, as discussed, guarantees an asymptotic advantage of our hybrid algorithm over a purely classical approach. This is one of our main results, and while the analytic estimate may miss some features, it is also (at least qualitatively) strongly supported by explicit computational experiments, presented below. In the future, it would be interesting to explore how these arguments generalise to other situations, where, e.g., the width of the minima itself scales with *M* and where subtler effects could appear, possibly still allowing the hybrid algorithm to outperform the classical one.

Our algorithm is implemented in Python, with the quantum part of the algorithm based on Qiskit. We performed the simulations on a noiseless emulator of Qiskit. We assume that it is possible to compute the function *f* on a quantum computer. In the experiments, we simply flip the phase of the target points *x* that satisfy f(x)<t, to implement the oracle for a threshold *t*.

In the BBW algorithm that we use for the quantum runs, we choose the parameter λ=8/7, as suggested in [11]. We terminate the cycle when the total number of queries becomes larger than $3\cdot 2.46\sqrt{cM}$ (the expected number of queries to reach the minimum value in the BBW approach described in Algorithm 1 is $2.46\sqrt{cM}$). The coefficient 3 was chosen empirically, after numerical examinations (see Section 3.2), so that the algorithm finds the global minimum with high probability.

### 3.2. Experimental Results

All tests were performed on a machine with the following characteristics:

- CPU: AMD Ryzen 7 PRO 8840U
- RAM: 32 GiB
- OS: Linux, kernel: 6.18.22-gentoo
- Python: 3.13.12
- marimo: 0.23.3
- numpy: 2.4.4
- pandas: 2.3.3
- plotly: 6.7.0
- qiskit: 2.2.3

The source code is available on request.

Functions.

For the primary tests, we use the one-dimensional Rastrigin function defined in Section 2.5. Since we need functions with different numbers of minima, we take the range of *x* to be $\left[-\frac{M-1}{2};\frac{M-1}{2}\right]$ to get a function with *M* minima. We also ensure that M<πA/2, because the Rastrigin function has a finite number of minima. A random constant shift is added to the argument of the Rastrigin function, i.e., ${\tilde{f}}_{z}\left(x\right)=f_{R1}\left(x-z\right)$, as a way to introduce some random noise and make the position of the true minimum an unknown point rather than at zero. An additional example is also considered involving random sine sum functions defined in Section 2.6 (corresponding details are considered below): $f\left(x\right)=\sum_{i=1}^{K}a_{i}\cos\left(\omega_{i}x+b_{i}\right).$ Recall that these functions are periodic, with a period of 2π. To obtain a function with *M* minima, we use the highest frequency $\omega =\max_{i}\left\{\omega_{i}\right\}$ to get an upper bound on the number of minima per period and take the range of *x* to be [0;2πM/ω]. Unless otherwise stated, analyses below concern the Rastrigin function.

Choice of *c*.

For the proposed algorithm, it is crucial to choose the correct discretisation. We outlined previously that we take it to contain cM points, with *c* being a key parameter. If the chosen value of *c* is too small, the algorithm could miss the neighbourhood of the true global minimum; if *c* is too large, we would need a very large number of quantum iterations, and there would be no advantage compared to the classical case (except at very large values of *M*). Our hybrid algorithm first uses quantum search to identify a suitable grid point, ideally corresponding to the minimum value amongst the points in the grid, and then runs Newton–Raphson’s method starting from that point. In order to choose the best grid size, we model the process with different values of *c*: we sample cM points, find the minimum value among them, and record whether Newton–Raphson’s method finds the global minimum starting from that point. For each value of *c* and each number of minima *M*, we performed 100 experiments. The resulting rate of success for the searches (i.e., the proportion of searches which identify the global minimum) for the Rastrigin-1D function is reported in Figure 1.

As expected, the proportion of successful searches increases with higher values of *c*. At around c=24, all searches become successful; therefore, we can choose c>24 to further examine the algorithm.

When the number of points is not equal to some power of two, i.e., $2^{k-1}<cM<2^{k}$ for some *k*, Grover’s algorithm requires k=⌈log(cM)⌉ qubits rather than log(cM), and the running time of the algorithm is larger than the naive estimate. To avoid these discretisation artefacts, we make the grid size equal to some power of 2, that is, we choose *M* to be a power of 2 and the value $c_{\mathrm{opt}}=32$.

Hybrid algorithm simulation.

The next series of experiments evaluates how many function evaluations are required with the value $c_{\mathrm{opt}}=32$ that we chose; results are shown in Figure 2. The graph shows the number of queries for purely classical Newton–Raphson’s method and for the hybrid method. For each number of minima *M*, we considered 100 function instances (i.e., Rastrigin functions with *M* minima and some random shift), and for each function instance we performed 10 repetitions, as a way to randomise the choice of the starting point for the Newton–Raphson method and to address measurement noise for the hybrid method (since the quantum part of the algorithm produces output from measurements, so that this output is probabilistic). Each point in the graph represents an average across 100×10=1000 examinations.

As we can see, the hybrid algorithm starts to outperform the classical one at around M=32. In the present series of experiments, we ran the hybrid algorithm until it found the global minimum. Of course, in real-world scenarios, the global minimum would not be known beforehand, so we have to set a termination condition based on the number of queries made. We can observe that 98.9% of all runs make fewer than $3\cdot 2.46\sqrt{cM}$ queries; therefore, in the next experiment series, which mimics more closely such real-world scenarios, the hybrid algorithm terminates after $3\cdot 2.46\sqrt{cM}$ total queries to the oracle.

Testing the termination condition.

The final series of experiments tests the hybrid algorithm in a scenario where the global minimum is not known, and the termination condition uses only the number of queries made. For each number of minima *M*, we selected 20 function instances, and for each function instance we performed 10 repetitions. Figure 3 and Table 2 show, correspondingly, how many iterations were performed by the hybrid algorithm and in how many cases it succeeded in finding the global minimum. The minimal ratio is 95% for a function with 80 minima, meaning that one in every twenty runs ends with a point that is not the global minimum. As in the previous series, the hybrid algorithm starts outperforming the classical one after M≈32.

Based on the results of these experiments, we propose using the following termination condition: the total number of queries should be bounded by $3\cdot 2.46\sqrt{c_{\mathrm{opt}}M}$, where $c_{\mathrm{opt}}=32$ is chosen in the previous paragraph and *M* is the expected number of function minima.

Applicability of the method to another class of functions.

The Rastrigin function is an interesting case for optimisation. We next consider in less detail another class of functions, random sine sum functions as defined in Section 2.6.

There are three purposes to this example. First, we illustrate the applicability of the hybrid method with another class of functions. Second, it is shown that the method itself works in this more general case, as demonstrated by the following Figure 4 and Figure 5. Finally, the example shows how the efficiency of the hybrid method depends on the properties of the function, including density of minima–the application with $\omega_{\max}=40$ shows the hybrid method is generally superior, while for $\omega_{\max}=80$, the hybrid method also shows an advantage, but across a smaller part of the investigated range regarding the number of minima (as elsewhere, algorithm performance is measured in terms of function queries).

The graphs for these experiments are less smooth and have larger confidence intervals than those for the Rastrigin function experiments. This behaviour could be explained by the fact that the random sine sums are less “structured” than the Rastrigin function. To achieve the same confidence interval as for the Rastrigin function experiments, many more repetitions would be required; still, we think the observed patterns in the results are indicative enough.

### 3.3. Theoretical Analysis of the Hybrid Algorithm

We provide some formalisation of the conditions that enable the hybrid algorithm to succeed. The following theorem is fairly straightforward, and the quantities used (radius of the convergence region and separation between function minima) cannot be easily computed for all functions. Nevertheless, this theorem represents an initial step towards a comprehensive analysis of the choice of discretisation for applying quantum algorithms to continuous problems.

**Theorem** **1.** *Consider a three-times-differentiable function f:[a,b]→R such that $|f^{\prime }\left(x\right)|<C_{1}$, $|f^{\prime \prime }\left(x\right)|<C_{2}$, and $|f^{\left(3\right)}\left(x\right)|<C_{3}$ for all x∈[a,b]. Suppose that there exists a constant R>0 such that every local minimum $z_{i}$ of f (i∈{1,…,M}) has a region of convergence of radius $r_{i}>R$. Suppose also that the global minimum*

$$z=z_{k}=\underset{z_{i},i\in \left\{1,\ldots ,M\right\}}{\mathrm{argmin}}f$$

*is separated from other minima by δ: for any i≠k, $f\left(z_{i}\right)-f\left(z\right)>\delta$.*

*Let $x_{i}=a+ih$, where i∈{0,…,cM−1} and h=(b−a)/(cM), be a grid of cM equidistant points on [a,b).*

*Suppose that $0<h<2\min\left\{R,\sqrt{\frac{6\delta -C_{3}R^{3}}{3C_{2}}}\right\}$ and $C_{3}R^{3}<6\delta$.*

*Then, the hybrid algorithm succeeds with probability at least 2/3.*

Note that the existence of a constant *R* is equivalent to the function having a finite number of local minima.

**Proof.**  The hybrid algorithm succeeds if Grover’s algorithm finds a grid point, and Newton’s method starting from this point identifies the global minimum. Since Grover’s algorithm finds the minimum among the grid points with constant probability 2/3, this is equivalent to the following conditions:

(1) there is at least one grid point $x_{i}$ in the region of convergence of the global minimum,
(2) the grid point $x_{k}$ with a minimal function value in the region of convergence of the global minimum has a smaller function value than other grid points.

Each region of convergence has 2R/h grid points, so there is at least one point in each region of convergence.

Using Taylor expansion around the global minimum *z*, we can write:

$$f\left(x\right)=\sum_{k=0}^{2}\frac{f^{\left(k\right)}\left(z\right)}{k!}{\left(x-z\right)}^{k}+R_{2}\left(x\right),$$

where $R_{2}\left(x\right)$ is the remainder term. By Taylor’s theorem, the remainder term is upper bounded by

$$|R_{2}\left(x\right)|\leq C_{3}\frac{{\left|x-z\right|}^{3}}{3!}\leq C_{3}\frac{R^{3}}{3!}$$

for all x∈(z−R,z+R).

Therefore, we have

$$\left|f\left(x\right)-f\left(z\right)|\leq \right|f^{\prime }\left(z\right)||x-z|+\frac{1}{2}\left|f^{\prime \prime }\left(z\right)\right|{\left(x-z\right)}^{2}+C_{3}\frac{R^{3}}{3!}.$$

Let $x_{0}$ be a grid point closest to the global minimum. Clearly, $|x_{0}-z|<h/2$ and $f^{\prime }\left(z\right)=0$, so we get

$$|f\left(x_{0}\right)-f\left(z\right)|\leq \frac{C_{2}}{2}{\left(\frac{h}{2}\right)}^{2}+\frac{C_{3}R^{3}}{6}.$$

Consider any other minimum $z_{j}$. The values of grid points $x_{j}$ in its region of convergence are larger than the value at $z_{j}$: $f\left(x_{j}\right)>f\left(z_{j}\right)$. Therefore, it is sufficient to prove that $f(x_{0})$ is less than $f(z_{j})$. We have:

$$f\left(z_{j}\right)-f\left(x_{0}\right)=f\left(z_{j}\right)-f\left(z\right)+f\left(z\right)-f\left(x_{0}\right)>\delta -\left(f\left(x_{0}\right)-f\left(z\right)\right)\geq \delta -\frac{C_{2}h^{2}}{8}-\frac{C_{3}R^{3}}{6}.$$

By requiring the r.h.s. to be positive, we obtain the following quadratic inequality in *h*:

$$\frac{C_{2}h^{2}}{8}+\frac{C_{3}R^{3}}{6}<\delta .$$

Solving it, we obtain:

$$h<2\sqrt{\frac{6\delta -C_{3}R^{3}}{3C_{2}}}$$

when $C_{3}<\frac{6\delta }{R^{3}}$. □

## 4. Conclusions

An important focus of research in quantum computing has been the identification of quantum algorithms which are guaranteed to outperform the best relevant classical ones, examples being Shor’s algorithm for the factorisation problem [18] and Grover’s algorithm for unstructured search [1]. While such demonstrations have had a foundational role in this research area and have produced undeniable benefits, it could be argued that there is also merit in considering a combination of classical and quantum algorithms, depending on the problem structure. We attempted to provide a preliminary investigation of this approach in the context of function optimisation (finding the global minimum of a function). In general, one may expect that the more random a function, the more suitable it would be to optimise assuming no structure–that is, employing Grover’s unstructured search; conversely, the more structured a function, the more we might benefit from classical algorithms, such as gradient descent or Newton–Raphson. What about functions which are ‘intermediate’ between these extreme (and impractical) cases of complete randomness and straightforward structure (e.g., a single global minimum)? There would be many functions of this form.

Informally, our proposal is that hybrid algorithms, composed of a quantum (Grover’s) regime and a classical (such as Newton–Raphson) regime, allow an ideal division of labour, in terms of the former addressing the unstructured part of a problem and the latter the structured part. An initial investigation of how quantum and classical algorithms can work in synergy for optimisation was by Lara et al. [12]. We built on the same broad idea, namely that Grover unstructured search can help get the optimisation process close enough to the global minimum, at which point, if the classical algorithm takes over, any subsequent computation is very efficient.

Specifically, the algorithm of [12] alternates repeatedly between quantum and classical stages: after a classical local optimisation step, Grover adaptive search is used again to escape the current local minimum and identify a potentially better region, and this cycle is iterated several times. By contrast, our algorithm uses the quantum stage only once, at the beginning, to locate a grid point in the vicinity of the global minimum, and then hands the problem over to Newton–Raphson for the final continuous refinement. This simpler model lets us focus directly on the discretisation problem and on a fair comparison with a purely classical baseline, which were not the main focus of the earlier hybrid proposal.

In this work, we aimed to provide an existence proof that there are cases when hybrid algorithms can have some advantage over classical ones. So, for our main example, we tried to identify a family of functions that exemplifies the kind of problem that should be best suited to our hybrid algorithm: a function with a global minimum ‘hidden’ amongst many local ones. The one-dimensional Rastrigin function, with many local minima and one true global minimum, offers the kind of landscape in which a classical local optimisation method is frequently trapped. Across the numerical experiments, the hybrid algorithm shows the expected $\sqrt{M}$ query growth, compared with approximately linear growth for repeated Newton–Raphson, and it succeeds in finding the global minimum in 95.0–100.0% of runs under the chosen termination rule. Results with the random sine sum functions also support the hybrid method. These results do not establish that the hybrid approach is preferable in general, but they do identify a clear and practically relevant regime in which a quantum–classical hybrid can deliver a genuine empirical advantage over a purely classical strategy.

There are several limitations to the present work. First, regarding the applicability of the hybrid algorithm, we have to assume that any target function would belong to a particular class with properties that we have some knowledge of. For example, the practical application of our method could proceed along the following lines: select several random functions from a class of interest; analyse them through conventional methods; count how many local minima they have. Then, the discretisation of an unknown target function from this class could be guided by this information. These requirements clearly restrict the applicability of the proposed method.

Second, the results presented here, which focus on specific cases, do not ‘prove’ the general effectiveness of hybrid algorithm proposals, whether ours or other related ones, such as Lara et al.’s [12]. An important objective for future work is to extend the testing of hybrid algorithms to a broader range of functions, as a way to characterise the scope of applicability of hybrids versus pure alternatives (whether classical or quantum), especially in practical cases, such as neural network training. More generally, it would be desirable to develop formal tests for when hybrids would be suitable and formal results concerning their effectiveness.

Third, a significant practical limitation of the proposed hybrid approach is that its quantum component may remain difficult to realise on near-term hardware. In particular, realistic noise levels and the limited number of available qubits are likely to make the implementation of sufficiently complex oracles for the function *f* challenging, especially when the function has a nontrivial structure or requires a large encoding overhead. These same hardware constraints also restrict the number of Grover iterations that can be performed reliably, potentially preventing the algorithm from reaching the regime in which its theoretical advantage becomes meaningful. As a consequence, although the hybrid scheme is conceptually attractive, currently available, empirically justified variational methods are likely to remain of greater practical utility in the short term, since they are typically more resilient to noise and better suited to the capabilities of NISQ devices.

If these limitations can be overcome, it would be interesting to explore possible applications of hybrid algorithms, such as high-dimensional non-convex optimisation problems, where classical methods are prone to becoming trapped in local minima, potentially including the training landscapes of deep neural networks or energy minimisation in molecular structure prediction. In these domains, the ability to combine a global search mechanism (Grover) with a fast local refinement step (Newton–Raphson) might offer an advantage over purely classical methods (such as gradient descent with random restarts) or purely quantum ones.

Moreover, theoretically, an interesting motivation for hybrid algorithms structured so that there is a single quantum regime to start with, followed by a classical process, relates to cognitive psychology. Briefly, as far as we know, brain neurophysiology is classical [19]. Nevertheless, there are some hints that classical neurophysiology may not be the whole story. First, the brain’s energy output seems very low, relative to what it accomplishes [20]. Moreover, there have been some hypotheses that there are sufficiently insulated structures in the brain to allow quantum computation, at least over short or very short periods of time (e.g., [21,22,23]). Second, there are many cognitive problems which can be solved at seemingly instantaneous speed, such as categorisation (an assignment of a new instance to an existing category, amongst as many as 50,000 concepts that an adult person has). Such problems posit cognitive mechanisms which defy plausibility given classical computation (for an overview of categorisation, see [24]). One could speculate that the apparent efficiency of cognitive computation might be partly due to a synergy between (most likely extremely brief) quantum computation and classical computation. Clearly, such a possibility is currently highly speculative.

Overall, one may expect that a more detailed investigation of the capabilities of hybrid algorithms may eventually turn out to shed some light on the above questions. In this paper, despite a number of limitations, we hope to have provided some promising results regarding the potential of hybrid algorithms, which will stimulate further interest. 

## Figures and Tables

**Figure 1:**
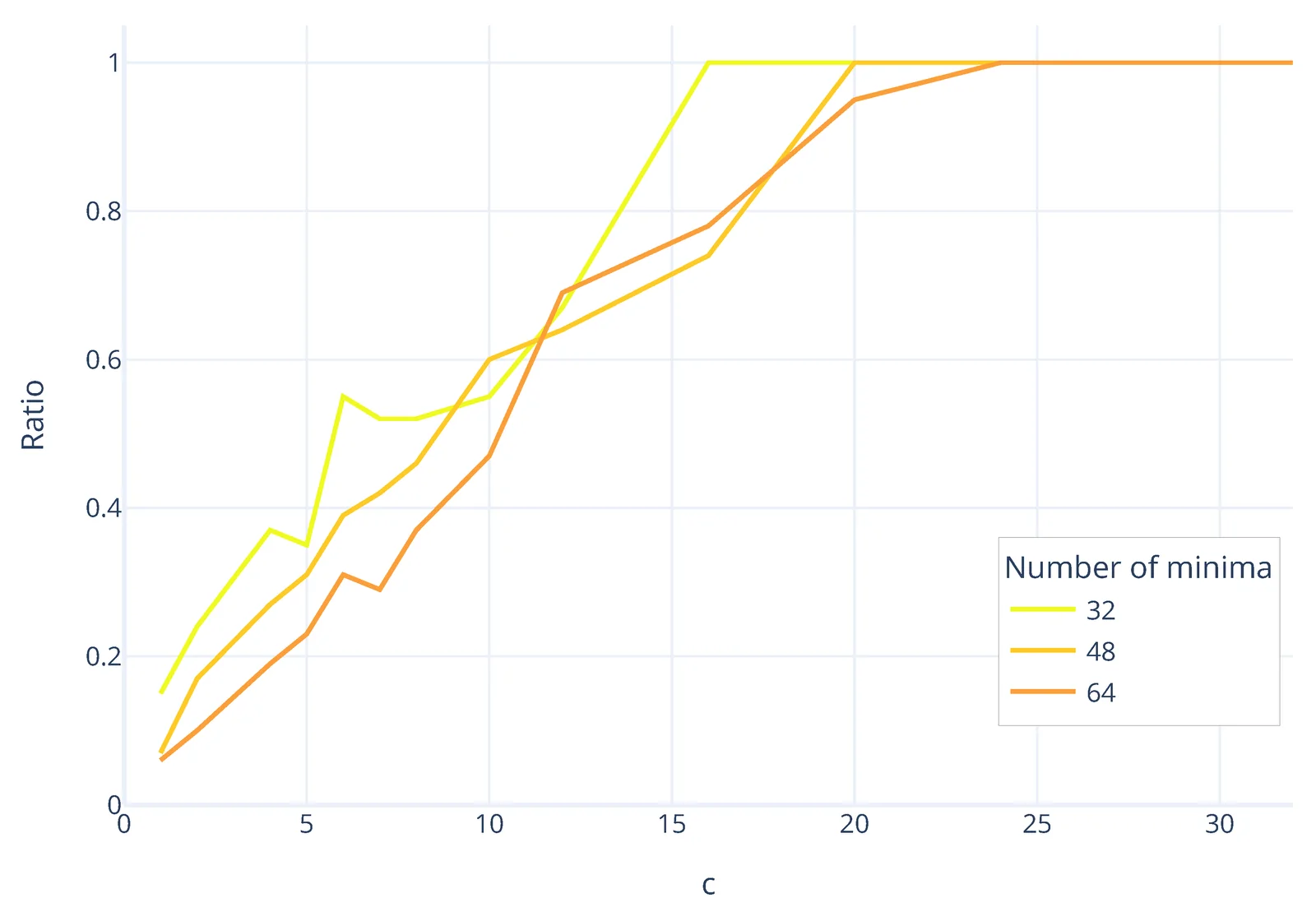
Success rate for finding the true minimum with different grid parameters *c*, for the Rastrigin-1D function. Colours denote functions with different numbers of minima.

**Figure 2:**
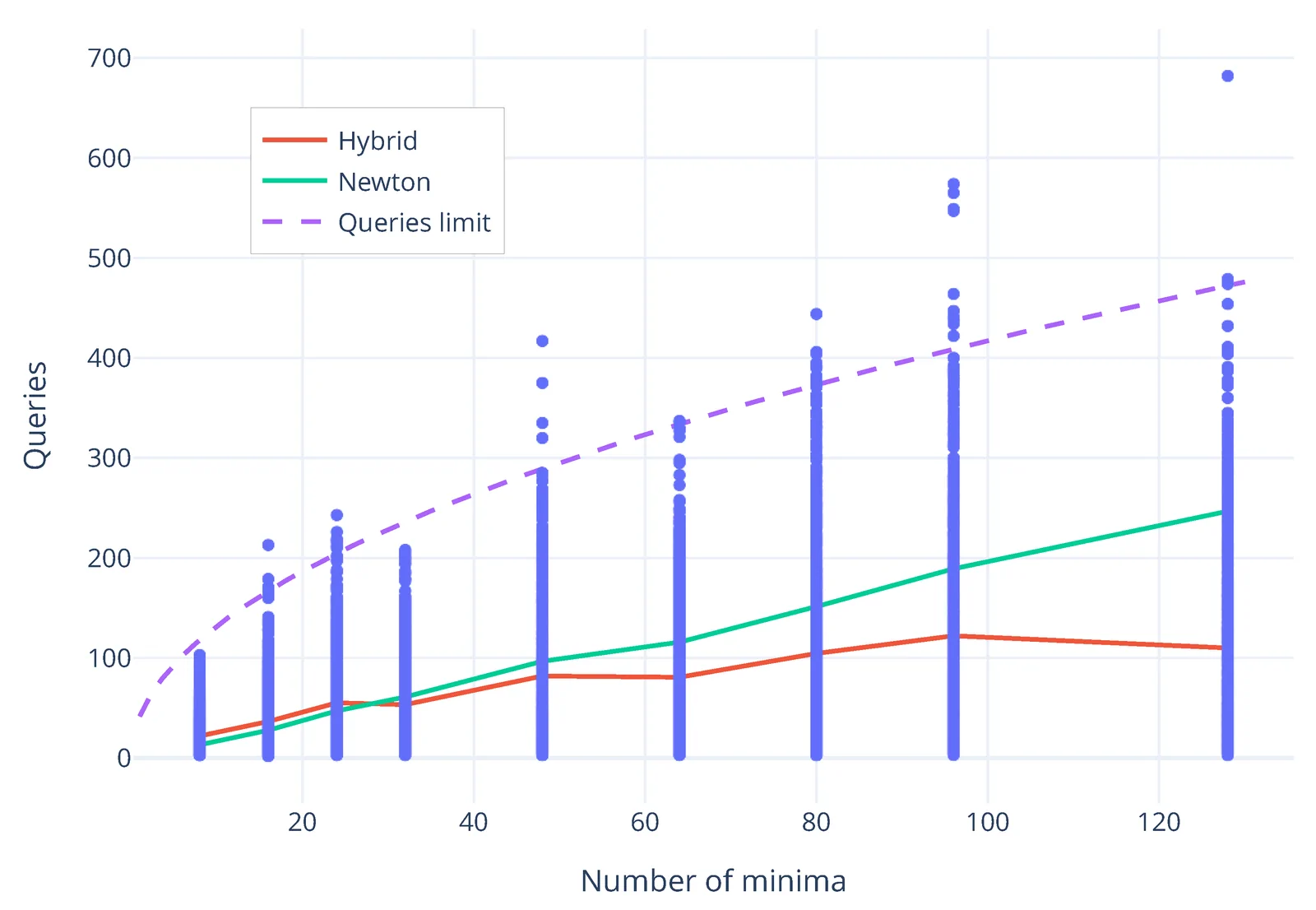
Queries (function evaluations) of the pure Newton–Raphson method (Newton) compared with the proposed hybrid algorithm for the Rastrigin-1D function. Each blue point denotes a hybrid algorithm application for some function instance. Red and green lines report an average number of queries for the hybrid and classical methods, respectively. The dashed purple line demonstrates the chosen termination condition for the hybrid algorithm.

**Figure 3:**
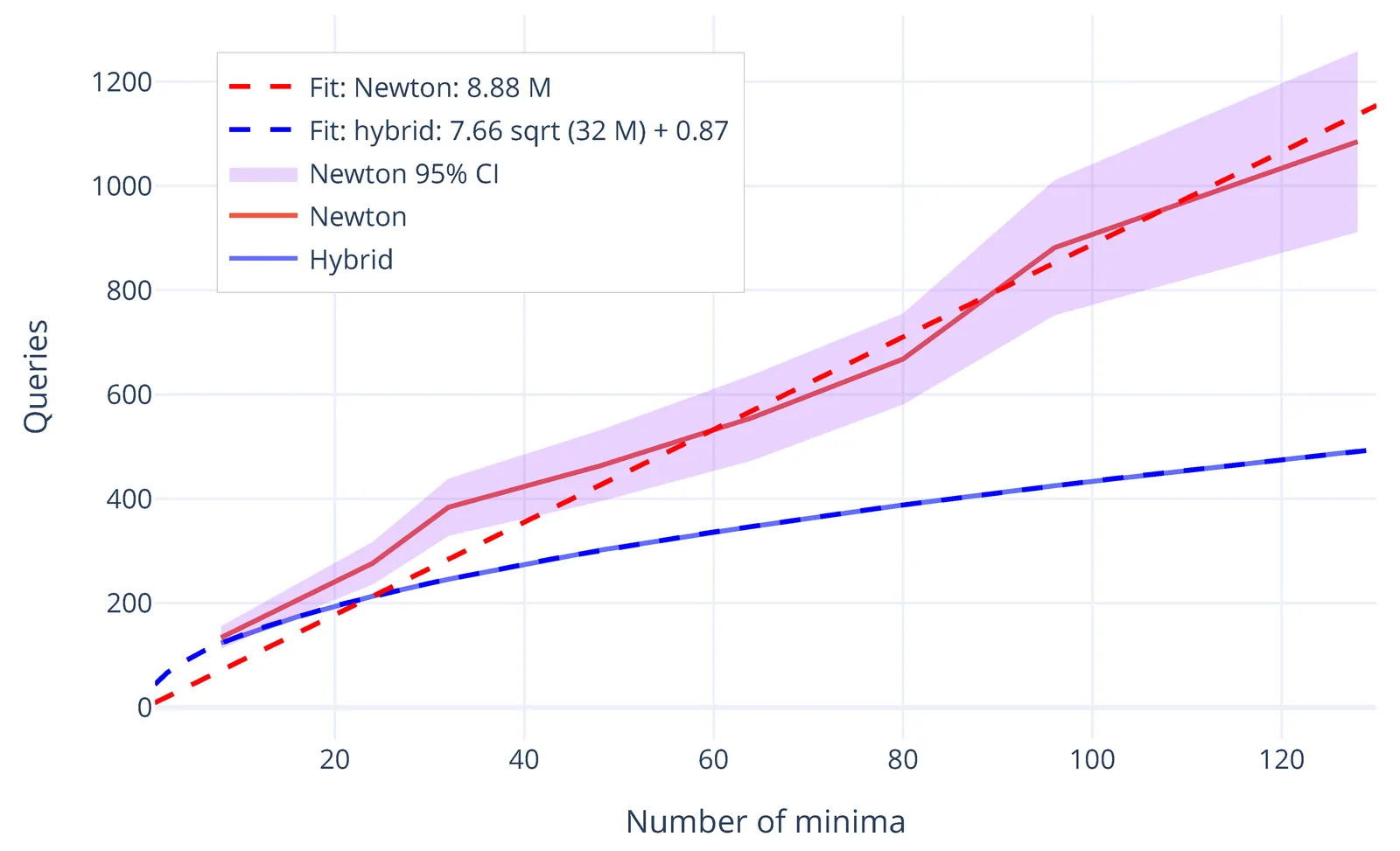
Queries (function evaluations) of the pure Newton–Raphson method (Newton) compared with the proposed hybrid algorithm for the Rastrigin-1D function. We see that the linear complexity for the classical method quickly overtakes the square-root one of the hybrid algorithm. The hybrid algorithm makes at most $3\cdot 2.46\sqrt{cM}$ queries.

**Figure 4:**
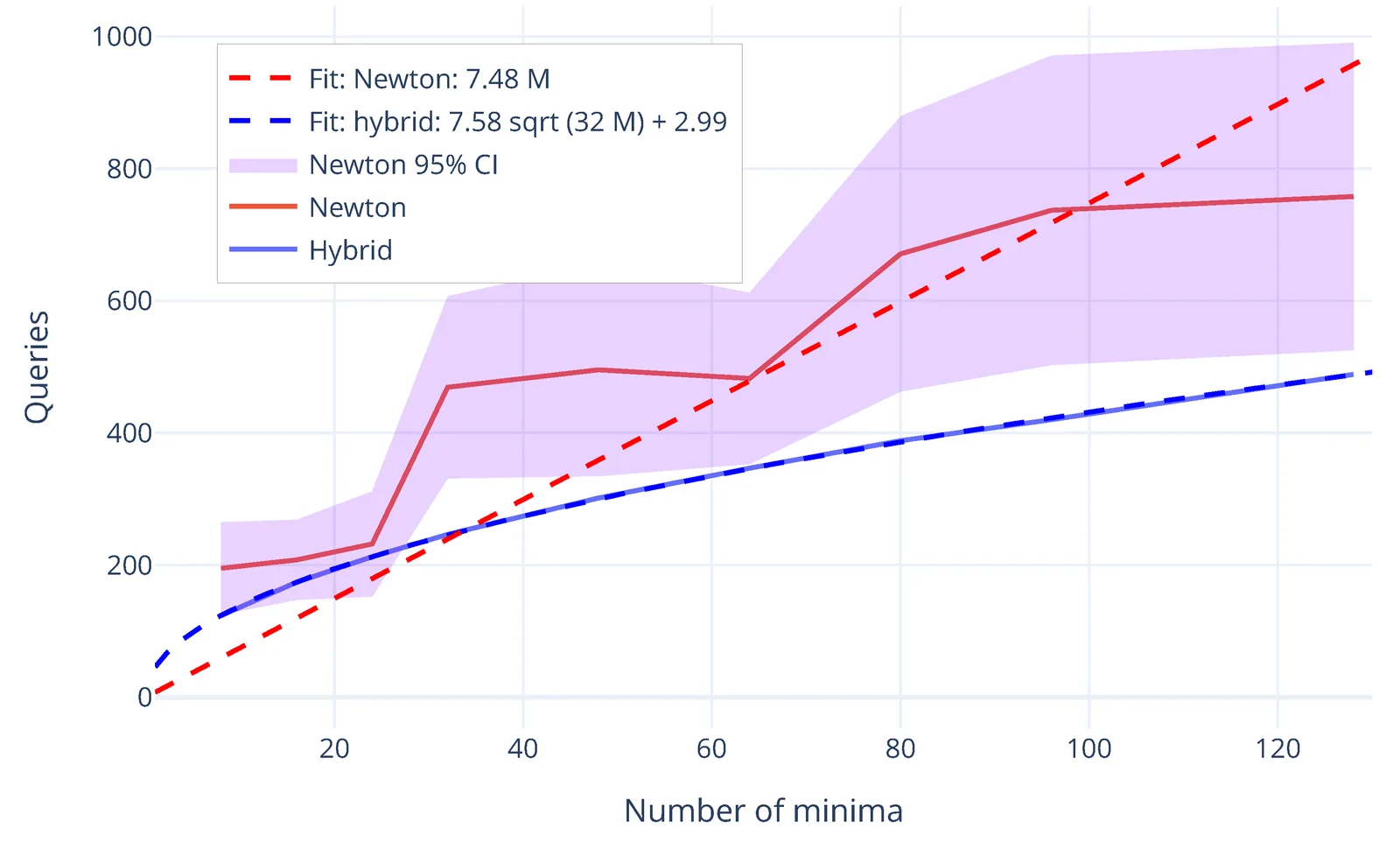
Number of queries for random sine sum function *f* with K=7 and $\omega_{\max}=40$. For each minimum, there are 10 random instances from the function class, and for each instance there are 5 repetitions.

**Figure 5:**
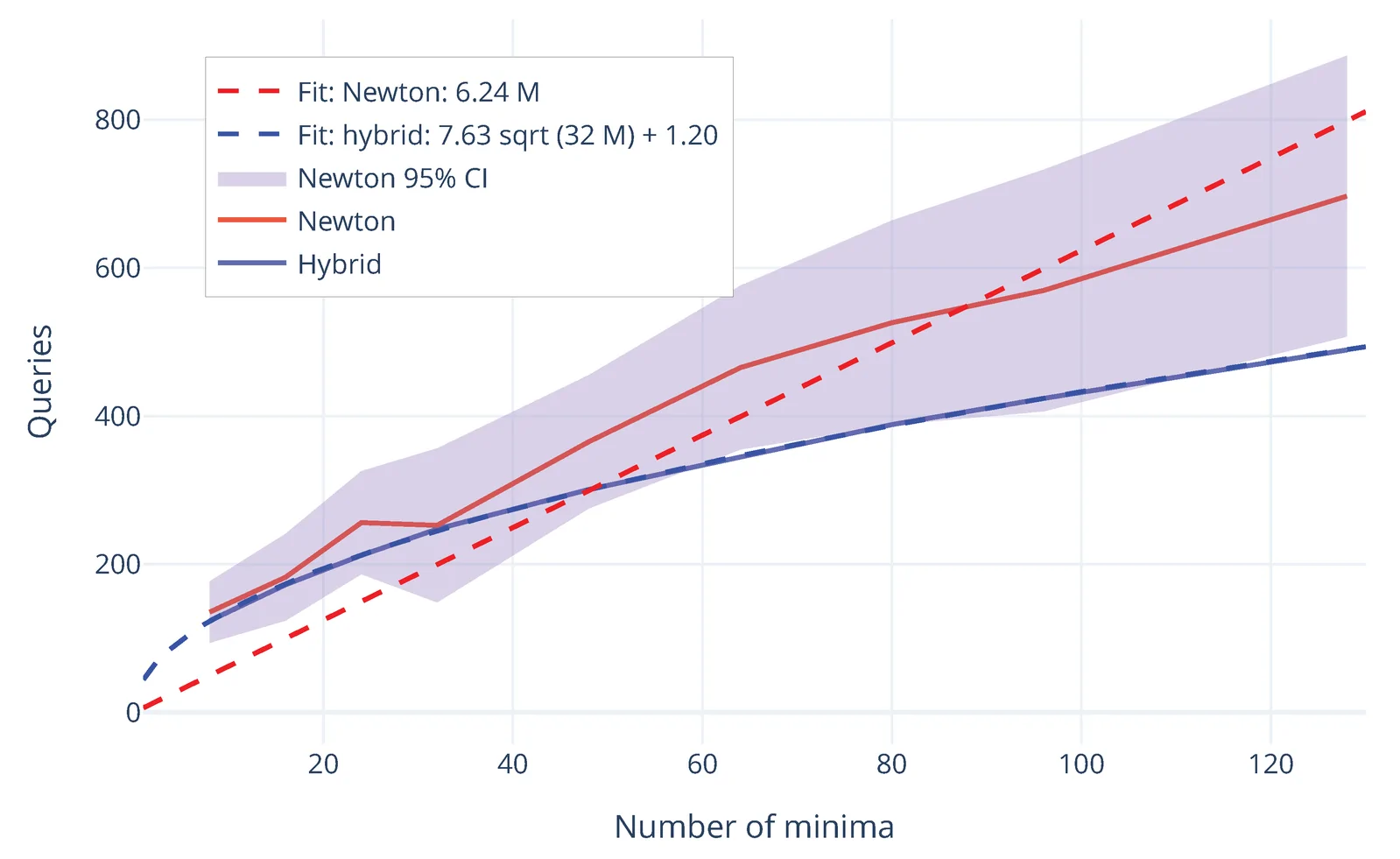
Number of queries for random sine sum function *f* with K=7 and $\omega_{\max}=80$. For each minimum, there are 10 random instances from the class, and for each instance there are 5 repetitions.

**Table 1:** Overview of quantum search and optimisation algorithms.

Algorithm	Oracle	Goal and Planned Runs	Schedule
Grover	$O_{a}:x\overset{?}{=}a$	number *t* of solutions is known, single run	$j=\lceil \frac{\pi }{4}\sqrt{N/t}\rceil$ rotations
Boyer et al.	$O_{a}:x\overset{?}{=}a$	number *t* of solutions is unknown, multiple runs	random number j∈{1,…,m−1} of rotations, rotations limit *m* is initialised with m:=1 and is updated as follows: $m:=\min\{6m/5,\sqrt{N}\}$
Durr and Hoyer	$O_{b}:x\overset{?}{<}b$	search for the global minimum	random number j∈{1,…,m−1} of rotations, rotations limit *m* is updated as follows: $m:=\min\{6m/5,\sqrt{N}\}$, algorithm stops after $22.5\sqrt{N}+1.4\log^{2}N$ total rotations
Baritompa et al. (BBW)	$O_{b}:x\overset{?}{<}b$	search for the global minimum	random number j∈{1,…,m−1} of rotations, rotations limit *m* is updated as follows: $m:=\min\{8m/7,\sqrt{N}\}$, algorithm’s expected running time is $2.46\sqrt{N}$ total rotations
Lara et al.	$O_{b}:x\overset{?}{<}b$	search for the global minimum	schedule is the same as in BBW, algorithm executes multiple Grover runs, with classical optimisation performed between them, updating the oracle threshold based on the classical optimisation results
This paper	$O_{b}:x\overset{?}{<}b$	search for the global minimum	schedule is the same as in BBW, algorithm runs the BBW search once and continues with the classical optimisation

**Table 2:** Ratio of global minima successfully found by the proposed hybrid algorithm for the Rastrigin-1D function. The hybrid algorithm makes at most $3\cdot 2.46\sqrt{cM}$ queries. Each row represents 20 different functions and 10 runs with different random seeds for each function.

Number of Minima	Ratio of Success
8	100.0%
16	99.0%
24	99.0%
32	99.5%
48	99.0%
64	99.5%
80	95.0%
96	99.5%
128	98.5%

## Data Availability

The original contributions presented in this study are included in the article. Further inquiries can be directed to the corresponding authors.
